# Retraction: Zhang et al. Functionalized Biomass Carbon-Based Adsorbent for Simultaneous Removal of Pb^2+^ and MB in Wastewater. *Materials* 2021, *14*, 3537

**DOI:** 10.3390/ma15020586

**Published:** 2022-01-13

**Authors:** Nannan Zhang, Nan Cheng, Qing Liu

**Affiliations:** 1 Modern Experiment Center, Harbin Normal University, Harbin 150025, China; 2 School of Chemical Engineering and Technology, Tianjin University, Tianjin 300350, China; Chengn1205@126.com

The journal retracts the article “Functionalized Biomass Carbon-Based Adsorbent for Simultaneous Removal of Pb^2+^ and MB in Wastewater” [1].

The journal was contacted by members of the author group of the publication, and following an investigation, in coordination with the Editor-in-Chief, major uncertainties concerning the integrity of the authors’ contributions and the scientific integrity of the publication became apparent.

The article is therefore retracted.

This retraction was approved by the Editor in Chief of the journal *Materials*.

The authors agreed to this retraction.
